# Pairing Optimization via Statistics: Algebraic Structure in Pairing Problems and Its Application to Performance Enhancement

**DOI:** 10.3390/e25010146

**Published:** 2023-01-11

**Authors:** Naoki Fujita, André Röhm, Takatomo Mihana, Ryoichi Horisaki, Aohan Li, Mikio Hasegawa, Makoto Naruse

**Affiliations:** 1 Department of Information Physics and Computing, Graduate School of Information Science and Technology, The University of Tokyo, 7-3-1 Hongo, Bunkyo-ku, Tokyo 113-8656, Japan; 2 Graduate School of Informatics and Engineering, The University of Electro-Communications, 1-5-1 Chofugaoka, Chofu-shi, Tokyo 182-8585, Japan; 3 Department of Electrical Engineering, Faculty of Engineering, Tokyo University of Science, 6-3-1 Niijuku, Katsushika-ku, Tokyo 125-8585, Japan

**Keywords:** pairing, optimization, matching, maximum weighted matching, heuristic algorithm

## Abstract

Fully pairing all elements of a set while attempting to maximize the total benefit is a combinatorically difficult problem. Such pairing problems naturally appear in various situations in science, technology, economics, and other fields. In our previous study, we proposed an efficient method to infer the underlying compatibilities among the entities, under the constraint that only the total compatibility is observable. Furthermore, by transforming the pairing problem into a traveling salesman problem with a multi-layer architecture, a pairing optimization algorithm was successfully demonstrated to derive a high-total-compatibility pairing. However, there is substantial room for further performance enhancement by further exploiting the underlying mathematical properties. In this study, we prove the existence of algebraic structures in the pairing problem. We transform the initially estimated compatibility information into an equivalent form where the variance of the individual compatibilities is minimized. We then demonstrate that the total compatibility obtained when using the heuristic pairing algorithm on the transformed problem is significantly higher compared to the previous method. With this improved perspective on the pairing problem using fundamental mathematical properties, we can contribute to practical applications such as wireless communications beyond 5G, where efficient pairing is of critical importance. As the pairing problem is a special case of the maximum weighted matching problem, our findings may also have implications for other algorithms on fully connected graphs.

## 1. Introduction

The procedure of generating pairs of elements among all entries of a given system often arises in various situations in science, technology, and economy [1,2,3,4,5,6,7]. Here, we call such a process pairing, and the number of elements is considered to be an even number for simplicity. One immediately obvious problem is that the number of pairing configurations grows rapidly with the number of elements. The number of possible pairings is given by (n−1)!!, where *n* indicates the number of elements in the system and !! is the double factorial operator. For example, when *n* is 100, the total number of possible pairings is on the order of $10^{78}$. Hence, finding the pairing that maximizes the benefit of the total system is difficult.

Notably, the pairing problem corresponds to the maximum weighted matching (MWM) problem on the complete graph. Multiple algorithms exist for solving the MWM problem [8,9,10,11,12,13,14,15]. In contrast to these conventional methods, we propose a heuristic and fast algorithm at the cost of some performance. The advantage of a fast heuristic algorithm is that it can be useful in environments where weights change dynamically or a quick pairing is required, such as in communications technology. A heuristic algorithm for the MWM problem using deep reinforcement learning was recently proposed by [16] with a similar goal. Furthermore, our research proposes algorithms that work under the limited observation constraint, which is explained later. In our previous study, we proposed an algorithm with a computational complexity of $O(n^{2})$ [17].

To the best of our knowledge, there is no exact algorithm that works on the order of $O(n^{2})$ for arbitrary weights. For example, Gabow [9] proposed a MWM algorithm with a computation time of $\left|E||V\right|+{\left|V\right|}^{2}\log\left|V\right|$, where *V* is a set of vertices and *E* is a set of edges. However, randomized or approximate algorithms can reduce computational time for some cases. For example, Cygan et al. [12] developed a randomized algorithm with a computation time of ${L|V|}^{\omega }$ for graphs with integer weights (ω<2.373 is the exponent of n×n matrix multiplication [18] and *L* is the maximum integer edge weight). Duan et al. [15] proposed an approximate algorithm achieving an approximation ratio of (1−ϵ)M with a computation time of $\left|E\right|\epsilon^{-1}\log\epsilon^{-1}$ for arbitrary weights and $\left|E\right|\epsilon^{-1}\log N$ for integer weights (ϵ is a positive arbitrary value and *M* is the maximum possible weight matching value). Here, |V|=n,|E|=n(n−1)/2. Here, we aim to improve our previous pairing problem result, i.e., to determine a higher-accuracy heuristic algorithm that works with $O(n^{2})$ computational complexity.

Note that the pairing problem should not be confused with the assignment problem, which is another special case of the MWM setting. The assignment problem requires the graph to be a weighted bipartite graph. Furthermore, in the assignment problem there are two classes of objects, where it is the goal to always match an object from the first class with an object from the second. However, in the pairing problem, there is only a single class of objects, and we allow any of them to be potentially paired with any other. The assignment problem is also related to the single-source shortest paths problem. Several well-known assignment algorithms [19,20,21] or single-source shortest paths algorithms [22] are known. For example, the Hungarian algorithm [19] solves the assignment problem $O(n^{3})$, the auction algorithm [20] works with parallelism and the Bellman–Ford algorithm runs with O(|V||E|) [22]. However, in this study, we consider a fully connected graph with an even number of elements, where the MWM problem cannot be solved by assignment problem algorithms.

An example of a pairing problem is found in a recent communication technology called non-orthogonal multiple access (NOMA) [23,24,25,26,27,28,29]. In NOMA, multiple terminals simultaneously share a common frequency band to improve the efficiency of frequency usage. The simultaneous use of the same frequency band causes interference in the signals from the base station to each terminal. To overcome this problem, NOMA uses a signal processing method called successive interference cancellation (SIC) [30] to distinguish individual channel information in the power domain, allowing multiple terminals to rely on the same frequency band. For simplicity, here we consider that the number of terminals that can share a frequency is given by two. Herein, the usefulness of the whole system can be measured by the total communication quality, such as high data throughput and low error rate, which depends crucially on the method of pairing.

The most fundamental parameter of the pairing problem is the merit between any two given elements, which we call individual compatibility, while the summation of compatibilities for a given pairing is called its total compatibility. The detailed definition is introduced below. Our goal is to derive pairings yielding high total compatibility.

In general, we do not need to assume that the individual compatibility of a pair is observable, i.e., only the total compatibility of a given pairing may be observed. Our previous study [17] divided the pairing problem into two phases. The first is the observation phase, where we observe total compatibilities for several pairings and estimate the individual compatibilities. The second is the combining phase, in which a search is performed for a pairing that provides high total compatibility. This procedure is referred to as pairing optimization. The search is based on the compatibility information obtained in the first phase. In [17], we show that the pairing optimization problem can be transformed into a travelling salesman problem (TSP) [31] with a three-layer structure, allowing us to benefit from a variety of known heuristics.

However, we consider that there is substantial room for further performance optimization. This study sheds new light on the pairing problem from two perspectives. The first is to clarify the algebraic structure of the pairing optimization problem. Because we care only about the total compatibility when all elements are paired, there are many compatibility matrices (defined in Section 2) that share the same total compatibilities. In other words, we can consider an equivalence class of compatibility matrices that yield the same total compatibilities and that cannot be distinguished if individual compatibilities are not measurable. We show that the compatibility matrices in each equivalence class have an invariant value.

Second, although any compatibility matrices in the same equivalence class theoretically provide the same total compatibility, the heuristic pairing optimization process can result in different total compatibility values. These differences are not caused by incomplete or noisy observations, but are due to the convergence properties of the heuristic pairing algorithms, which yield better results on some distributions than others. We examine how the statistics of the compatibility matrix affect the pairing optimization problem and propose a compatibility matrix that yields higher total compatibility after optimization. More specifically, we propose a transformation to the compatibility matrix that minimizes the variance of the elements therein, which we call the variance optimization. We confirmed numerically that enhanced total compatibility is achieved via the compatibility matrix after variance optimization. Furthermore, the proposed variance optimization algorithm may also be applicable when no observation phase is required, i.e., when the individual compatibilities are directly observable. In other words, there are cases where a compatibility matrix unsuitable for a heuristic combining algorithm can be converted to one that is easily combinable.

The remainder of this paper is organized as follows. In Section 2, we define the pairing optimization problem mathematically. Section 3 describes the mathematical properties of the equivalence class. Section 4 explains the concept of variance optimization and presents a solution by which it can be achieved. Section 5 presents results of numerical simulations of the proposed variance optimization. Furthermore, there are two optimization problems in this paper. The first is the pairing problem we aim to solve in Section 2.1. Second is the variance optimization which enables us to enhance the performance of the PNN+p2-opt algorithm in Section 4.2. Finally, Section 6 concludes the paper.

## 2. Problem Setting

In this section, we provide a mathematical definition of the pairing optimization problem that we address in this study, and define some of the mathematical symbols used in the following discussion. In addition, we explain the constraints applied to the pairing optimization problem.

### 2.1. Pairing Optimization Problem

Here, we assume that the number of elements is an even natural integer *n*, while the index of each element is a natural number between 1 and *n*. Parts of the pairing problem can be described elegantly in set theory, while others benefit from using matrix representations. We will use either, where appropriate. Here we use U(n) to denote the set of *n* elements:

(1) $$\begin{array}{c} U(n)\equiv \{i|i\in Z,1\leq i\leq n\}. \end{array}$$

Then, we define the set of all possible pairs for U(n) as P(n), which contains N(N−1)/2 pairs:

(2) $$\begin{array}{c} P(n)\equiv \{\{i,j\}|i,j\in U(n),i<j\}. \end{array}$$

To describe the compatibilities of these pairs, we now define a “compatibility matrix’’ *C* as follows:

$$\begin{array}{ccc} & & C\in R^{n\times n},\\ & & \forall \left\{i,j\right\}\in P\left(n\right),C_{i,j}=C_{j,i},\\ & & 1\leq i\leq n,C_{i,i}=0. \end{array}$$

The compatibility between elements *i* and *j* is denoted by $C_{i,j}\in R$. The matrix *C* is always symmetric and the major diagonal is zero, because pairing *i* and *j* does not depend on the order of elements and an element cannot be paired with itself. The set of all possible compatibility matrices is denoted as $\Omega_{n}$ when the number of elements is *n*. In other words, $\Omega_{n}$ is the set of all n×n symmetric distance matrices, or symmetric hollow matrices. To describe a pairing, i.e., which elements are paired together, we now define a pairing matrix $S\in R^{n\times n}$:

$$\begin{array}{ccc} & & \forall \left\{i,j\right\}\in P\left(n\right),S_{i,j}=S_{j,i}\;\mathrm{and}\;S_{i,j}\in \left\{0,1\right\},\\ & & 1\leq i\leq n,S_{i,i}=0,\\ & & \forall i,\sum_{j=1}^{n}S_{i,j}=1. \end{array}$$

*S* is symmetric, because pairing element *i* with *j* is equivalent to pairing *j* with *i*. The pairing matrix *S* is also hollow, because pairing *i* with itself is not allowed. Each row and column contains only a single non-zero element, as each element *i* can only be paired once. Therefore, a pairing matrix *S* is an n×n symmetric and hollow permutation matrix. We define the set of all pairing matrices S(n)≡{S} when the number of elements is *n*:

(3) $$\begin{array}{c} S\in S(n). \end{array}$$

To derive the set representation of a pairing, we introduce the map $f_{\mathrm{set}}$ as follows:

(4) $$\begin{array}{c} f_{\mathrm{set}}\left(S\right)\equiv \left\{\left\{i,j\right\}|i<j\;\mathrm{and}\;S_{i,j}=1\right\}. \end{array}$$

A function denoted by 〈X,C〉 is then defined as follows, using the Frobenius inner product ${\langle \cdot \rangle }_{F}$:

$$\begin{array}{ccc} & & C\in \Omega_{n},\\ & & X\in R^{n\times n},\\ & & \langle X,C\rangle =\frac{1}{2}{\langle X,C\rangle }_{F}. \end{array}$$

For a given compatibility matrix *C*, we call 〈S,C〉 for S∈S(n) the “total compatibility’’ for pairing *S*. This formulation is equivalent to the one used in our previous work [17], and corresponds to summing the individual compatibilities $C_{i,j}$ of the pairs defined by *S*:

$$\begin{array}{ccc} & & \langle S,C\rangle =\sum_{\left\{i,j\right\}\in f_{\mathrm{set}}\left(S\right)}C_{i,j}. \end{array}$$

For any given compatibility matrix *C*, the pairing optimization problem can then be formulated as follows:

$$\begin{array}{ccc} & & \max:\;\langle S,C\rangle ,\\ & & \mathrm{subject}\;\mathrm{to}:\;S\in S(n). \end{array}$$

### 2.2. Limited Observation Constraint

As briefly mentioned in Section 1, in practice there may often exist one more constraint on the pairing optimization problem. We will assume that initially we do not know each compatibility value. Moreover, we assume that only the value of total compatibility 〈S,C〉 for any pairing S∈S(n) is observable. We call this condition the “limited observation constraint’’.

Under this constraint, we must execute two phases, the “observation phase’’ and the “combining phase’’, as introduced in our previous study [17]. First, we estimate the ground-truth compatibility matrix $C^{g}$ through observations of the total compatibilities of several pairings in the observation phase. We denote the estimated compatibility matrix by $C^{e}$. Our previous work [17] calculated the minimum number of observations that are necessary for deducing $C^{e}$ and presents a simple algorithm for doing so efficiently.

## 3. Mathematical Properties of the Pairing Problem

In this section, we consider algebraic structures in the pairing problem. An equivalence relation is defined among compatibility matrices to construct equivalence classes. Then we show a conserved quantity within the equivalence class and that all members of the class yield the same total compatibility for any given pairing. Furthermore, the statistical properties of compatibility matrices are examined, forming the mathematical foundation of the variance optimization to be discussed in Section 4.

### 3.1. Adjacent Set

We define the adjacent set matrix $R_{i}\left(1\leq i\leq n\right)$ as follows:

(5) $$\begin{array}{ccc} & & R_{i}\in R^{n\times n},\\ & & {\left(R_{i}\right)}_{k,l}=\left\{\begin{array}{c} 1\;\mathrm{if}\;i\in \{k,l\}\;\mathrm{and}\;k\neq l\\ 0\;\mathrm{otherwise}\;. \end{array}\right. \end{array}$$

We can also describe $f_{\mathrm{set}}\left(R_{i}\right)$ as follows:

(6) $$\begin{array}{c} f_{\mathrm{set}}\left(R_{i}\right)=\left\{\{i,j\}|1\leq j\leq n,j\neq i\right\}. \end{array}$$

With these adjacent sets, the following theorem holds.

**Theorem** **1.** *$C\in \Omega_{n}$ is fully determined by {〈S,C〉∣S∈S(n)} and $\left\{\langle R_{i},C\rangle |1\leq i\leq n-1\right\}$.*

Note that $\langle R_{n},C\rangle$ is not included, i.e., only n−1 terms involving $R_{i}$ are needed. Here, we have chosen to exclude index *n* without loss of generality.

**Proof** **of** **Theorem** **1.** Our strategy to prove this involves calculating the dimension of the involved subspaces. First, we prove the equation

(7) $$\begin{array}{c} \mathrm{span}{\left\{S\right\}}_{S\in S(n)}\cap \mathrm{span}{\left\{R_{i}\right\}}_{1\leq i\leq n-1}=\left\{O_{n}\right\} \end{array}$$

where $O_{n}$ denotes the n×n zero matrix. Then, we focus on the following equation to check linear independence. Here, we number all pairings such as $S_{1},S_{2},\cdots S_{u}\cdots S_{(N-1)!!}$. We introduce the coefficients $a_{u}$ and $b_{v}$ and calculate the overlap of the spans:

(8) $$\begin{array}{ccc} & & 1\leq u\leq \left(n-1\right)!!,a_{u}\in R,\\ & & 1\leq v\leq n-1,b_{v}\in R,\\ & & \sum_{u=1}^{(n-1)!!}a_{u}S_{u}=\sum_{v=1}^{n-1}b_{v}R_{v}. \end{array}$$

We focus on the summation of the *k*th-column on both sides. Note that for every $S_{u}$ there is exactly one non-zero element in column *k*, while for $R_{v}$ there may be more than one if v=k and 1≤k≤n−1, or exactly one non-zero element otherwise. Then, the following equations hold:

When 1≤k≤n−1

(9) $$\begin{array}{c} \left(n-2\right)b_{k}+\sum_{l=1}^{n-1}b_{l}-\sum_{l=1}^{(n-1)!!}a_{l}=0. \end{array}$$

When k=n (because of our choice in formulating Theorem 1)

(10) $$\begin{array}{c} \sum_{l=1}^{n-1}b_{l}-\sum_{l=1}^{(n-1)!!}a_{l}=0. \end{array}$$

With Equations (9) and (10), $b_{k}=0\;\left(1\leq k\leq n-1\right)$ holds. This means that

(11) $$\begin{array}{ccc} & & \mathrm{span}{\left\{S\right\}}_{S\in S(n)}\cap \mathrm{span}{\left\{R_{i}\right\}}_{1\leq i\leq n-1}=\left\{O_{n}\right\}, \end{array}$$

(12) $$\begin{array}{ccc} & & \dim\;\mathrm{span}{\left\{R_{i}\right\}}_{1\leq i\leq n-1}=n-1. \end{array}$$

By our previous study [17],

(13) $$\begin{array}{c} \dim\;\mathrm{span}{\left\{S\right\}}_{S\in S(n)}=L_{\min}\left(n\right). \end{array}$$

Here, we denote $L_{\min}\left(n\right)\equiv \left(n-1\right)\left(n-2\right)/2$. By Equations (12) and (13), the following equation holds:

(14) $$\begin{array}{c} \dim\;\mathrm{span}{\left\{S\right\}}_{S\in S(n)}+\dim\;\mathrm{span}{\left\{R_{i}\right\}}_{1\leq i\leq n-1}=\dim\;\Omega_{n}. \end{array}$$

Therefore, by Equations (11) and (14),

(15) $$\begin{array}{c} \dim\;\mathrm{span}{\left\{S\right\}}_{S\in S(n)}\cup \mathrm{span}{\left\{R_{i}\right\}}_{1\leq i\leq n-1}=\dim\;\Omega_{n}. \end{array}$$

The pairing matrices *S* are a subset of $\Omega_{n}$. In addition, the adjacent set matrices $R_{i}$ are also a subset of $\Omega_{n}$. Therefore, the following equation holds:

(16) $$\begin{array}{c} \mathrm{span}{\left\{S\right\}}_{S\in S(n)}\cup \mathrm{span}{\left\{R_{i}\right\}}_{1\leq i\leq n-1}\subseteq \Omega_{n}. \end{array}$$

With Equations (15) and (16),

(17) $$\begin{array}{c} \mathrm{span}{\left\{S\right\}}_{S\in S(n)}\cup \mathrm{span}{\left\{R_{i}\right\}}_{1\leq i\leq n-1}=\Omega_{n}. \end{array}$$

That is, ${\left\{S\right\}}_{S\in S(n)}$ plus ${\left\{R_{i}\right\}}_{1\leq i\leq n-1}$ can construct $\Omega_{n}$. Finally, 〈S,C〉 is a linear transformation of *S* which comes from the property of the Frobenius inner product. Therefore, $C\in \Omega_{n}$ can be constructed as a linear combination of {〈S,C〉∣S∈S(n)} and $\left\{\langle R_{i},C\rangle |1\leq i\leq n-1\right\}$. Therefore, the theorem holds. □

**Corollary** **1.**
(18) $$\begin{array}{ccc} & & A,B\in \Omega_{n},\\ & & A=B\;if\;and\;only\;if\;\\ & & \forall S\in S(n),\\ & & \langle S,A\rangle =\langle S,B\rangle \;and\;1\leq i\leq n,\langle R_{i},A\rangle =\langle R_{i},B\rangle . \end{array}$$

This corollary is a special case of Theorem 1 because Equation (18) means that *A* and *B* have the same total compatibilities for all pairings and all adjacent sets.

Here, we present an example for Theorem 1 for the n=4 case to illustrate the relationship of the involved subspaces. We define the following $H_{i}$:

(19) $$\begin{array}{c} H_{i}=\left\{\begin{array}{c} \mathrm{span}{\left\{S\right\}}_{S\in S(n)}\;\mathrm{if}\;i=0,\\ \mathrm{span}\{R_{i}\}\;\mathrm{if}\;1\leq i\leq n-1. \end{array}\right. \end{array}$$

We represent $H_{i}$ as follows, where $D_{i,j}\in \Omega_{n}$ is defined as the n×n matrix whose (i,j)th element is 1 and all other elements are 0:

(20) $$\begin{array}{ccc} & & H_{i}=\left\{\begin{array}{c} \;\mathrm{if}\;i=0,\\ \left\{k_{1}\left(D_{1,2}+D_{3,4}\right)+k_{2}\left(D_{1,3}+D_{2,4}\right)+k_{3}\left(D_{1,4}+D_{2,3}\right)|k_{1},k_{2},k_{3}\in R\right\}\\ \;\mathrm{if}\;i=1,\\ \left\{k_{4}\left(D_{1,2}+D_{1,3}+D_{1,4}\right)|k_{4}\in R\right\}\\ \;\mathrm{if}\;i=2,\\ \left\{k_{5}\left(D_{2,1}+D_{2,3}+D_{2,4}\right)|k_{5}\in R\right\}\\ \;\mathrm{if}\;i=3,\\ \{k_{6}\left(D_{3,1}+D_{3,2}+D_{3,4}\right)|k_{6}\in R\}, \end{array}\right. \end{array}$$

(21) $$\begin{array}{ccc} & & \bar{H}=\left\{l_{i,j}D_{i,j}|1\leq i<j\leq n,l_{i,j}\in R\right\}. \end{array}$$

**Figure 1:**
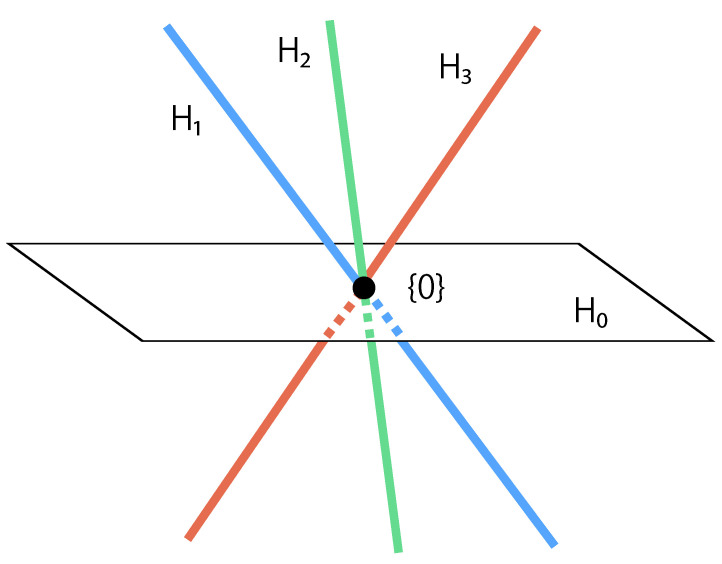
A schematic illustration of the relationship among $H_{0},H_{1},H_{2}$ and $H_{3}$.

The image of these spaces is represented in Figure 1. That is,

(22) $$\begin{array}{ccc} & & 0\leq i<j\leq n-1,i\neq j,H_{i}\cap H_{j}=\left\{O_{n}\right\}, \end{array}$$

(23) $$\begin{array}{ccc} & & \bar{H}=H_{0}\cup H_{1}\cup H_{2}\cup H_{3}. \end{array}$$

### 3.2. Equivalence Class

We define the relation ∼ as follows:

(24) $$\begin{array}{ccc} & & A,B\in \Omega_{n},\\ & & A∼B\;\mathrm{if}\mathrm{and}\mathrm{only}\mathrm{if}\;\forall S\in S(n),\langle S,A\rangle =\langle S,B\rangle . \end{array}$$

This represents an equivalence relationship between *A* and *B*, leading to the construction of an equivalence class.

Regarding this equivalence class, the following theorem holds:

**Theorem** **2.**
(25) $$\begin{array}{ccc} & & A,B\in \Omega_{n},\\ & & A∼B\;if\;and\;only\;if\;\\ & & \forall \{i,j\}\in P(n),\\ & & A_{i,j}-\frac{1}{n-2}\left(\langle R_{i},A\rangle +\langle R_{j},A\rangle \right)=B_{i,j}-\frac{1}{n-2}\left(\langle R_{i},B\rangle +\langle R_{j},B\rangle \right). \end{array}$$

*That is, for any matrix C in the equivalence class, the values given by the following are conserved.*

(26) $$\begin{array}{c} \forall \left\{i,j\right\}\in P\left(n\right),C_{i,j}-\frac{1}{n-2}\left(\langle R_{i},C\rangle +\langle R_{j},C\rangle \right). \end{array}$$

The matrix form of the conserved values is described in Appendix A.

**Proof** **of** **Theorem** **2.** First, we prove sufficiency. We assume that the following equation holds:

(27) $$\begin{array}{c} \forall \left\{i,j\right\}\in P\left(n\right),A_{i,j}-\frac{1}{n-2}\left(\langle R_{i},A\rangle +\langle R_{j},A\rangle \right)=B_{i,j}-\frac{1}{n-2}\left(\langle R_{i},B\rangle +\langle R_{j},B\rangle \right). \end{array}$$

With Equation (27), the following equation holds:

(28) $$\begin{array}{ccc} & & \sum_{\left\{i,j\}\in P(n\right)}\left\{A_{i,j}-\frac{1}{n-2}\left(\langle R_{i},A\rangle +\langle R_{j},A\rangle \right)\right\}\\ & & =\sum_{\left\{i,j\}\in P(n\right)}\left\{B_{i,j}-\frac{1}{n-2}\left(\langle R_{i},B\rangle +\langle R_{j},B\rangle \right)\right\}. \end{array}$$

Here, the left side can be calculated as follows because the number of pairs including element *k* in P(n) is n−1:

(29) $$\begin{array}{ccc} & & \sum_{\left\{i,j\}\in P(n\right)}\left\{A_{i,j}-\frac{1}{n-2}\left(\langle R_{i},A\rangle +\langle R_{j},A\rangle \right)\right\}\\ & = & \sum_{\left\{i,j\}\in P(n\right)}A_{i,j}-\frac{n-1}{n-2}\sum_{k=1}^{n}\langle R_{k},A\rangle \\ & = & \sum_{\left\{i,j\}\in P(n\right)}A_{i,j}-\frac{n-1}{n-2}\sum_{k=1}^{n}\sum_{l\neq k}A_{k,l}\\ & = & \sum_{\left\{i,j\}\in P(n\right)}A_{i,j}-\frac{2(n-1)}{n-2}\sum_{\left\{k,l\}\in P(n\right)}A_{k,l}\\ & = & -\frac{n}{n-2}\sum_{\left\{i,j\}\in P(n\right)}A_{i,j}. \end{array}$$

Using Equation (29), Equation (28) is transformed into the following:

(30) $$\begin{array}{c} -\frac{n}{n-2}\sum_{\left\{i,j\}\in P(n\right)}A_{i,j}=-\frac{n}{n-2}\sum_{\left\{i,j\}\in P(n\right)}B_{i,j}. \end{array}$$

Therefore,

(31) $$\begin{array}{c} \sum_{\left\{i,j\}\in P(n\right)}A_{i,j}=\sum_{\left\{i,j\}\in P(n\right)}B_{i,j}. \end{array}$$

The following equation holds for any pairing *S* by Equation (27):

(32) $$\begin{array}{ccc} & & \sum_{\left\{i,j\right\}\in f_{\mathrm{set}}\left(S\right)}\left\{A_{i,j}-\frac{1}{n-2}\left(\langle R_{i},A\rangle +\langle R_{j},A\rangle \right)\right\}\\ & & =\sum_{\left\{i,j\right\}\in f_{\mathrm{set}}\left(S\right)}\left\{B_{i,j}-\frac{1}{n-2}\left(\langle R_{i},B\rangle +\langle R_{j},B\rangle \right)\right\}. \end{array}$$

Here, the following equation holds. Note that {i,j} belongs to $f_{\mathrm{set}}\left(S\right)$; hence, $\langle R_{k},A\rangle$ appears only once and all indexes *k* ranging from 1 to *n* appear over the summation:

(33) $$\begin{array}{ccc} \sum_{\left\{i,j\right\}\in f_{\mathrm{set}}\left(S\right)}\left(\langle R_{i},A\rangle +\langle R_{j},A\rangle \right) & = & \sum_{k=1}^{n}\langle R_{k},A\rangle \\ & = & \sum_{k=1}^{n}\sum_{l,l\neq k}A_{k,l} \end{array}$$

(34) $$\begin{array}{ccc} & = & 2\sum_{\left\{k,l\}\in P(n\right)}A_{k,l}. \end{array}$$

For *B*, the following equation also holds:

(35) $$\begin{array}{ccc} \sum_{\left\{i,j\right\}\in f_{\mathrm{set}}\left(S\right)}\left(\langle R_{i},B\rangle +\langle R_{j},B\rangle \right) & = & \sum_{k=1}^{n}\langle R_{k},B\rangle \end{array}$$

(36) $$\begin{array}{ccc} & = & 2\sum_{\left\{k,l\}\in P(n\right)}B_{k,l}. \end{array}$$

Using these transformations, Equation (32) is transformed as follows:

(37) $$\begin{array}{c} \langle S,A\rangle -\frac{2}{n-2}\sum_{\left\{k,l\}\in P(n\right)}A_{k,l}=\langle S,B\rangle -\frac{2}{n-2}\sum_{\left\{k,l\}\in P(n\right)}B_{k,l}. \end{array}$$

With Equation (31),

(38) $$\begin{array}{ccc} & & \langle S,A\rangle =\langle S,B\rangle . \end{array}$$

Then, A∼B holds.

Second, we prove the necessity. We assume that A∼B holds. We define $A^{*}\in \Omega_{n}$ as follows:

(39) $$\begin{array}{c} A_{i,j}^{*}\equiv \frac{1}{n-2}\left(\langle R_{i},A\rangle +\langle R_{j},A\rangle \right)+B_{i,j}-\frac{1}{n-2}\left(\langle R_{i},B\rangle +\langle R_{j},B\rangle \right). \end{array}$$

By Equations (33), (35) and (39),

(40) $$\begin{array}{ccc} \forall S\in S\left(n\right),\langle S,A^{*}\rangle & = & \sum_{\left\{i,j\right\}\in f_{\mathrm{set}}\left(S\right)}A_{i,j}^{*}\\ & = & \langle S,B\rangle +\frac{1}{n-2}\sum_{i=1}^{n}\langle R_{i},A\rangle -\frac{1}{n-2}\sum_{i=1}^{n}\langle R_{i},B\rangle . \end{array}$$

We derive the relationship between $\sum_{i=1}^{n}\langle R_{i},A\rangle$ and $\sum_{S\in S(n)}\langle S,A\rangle$ here in order to transform Equation (40). By Equation (34),

(41) $$\begin{array}{c} \sum_{i=1}^{n}\langle R_{i},A\rangle =2\sum_{\left\{i,j\}\in P(n\right)}A_{i,j}. \end{array}$$

For $\sum_{S\in S(n)}\langle S,A\rangle$, we focus on the fact that the number of appearances of $A_{i,j}$ is (n−3)!!,

(42) $$\begin{array}{c} \sum_{S\in S(n)}\langle S,A\rangle =\left(n-3\right)!!\sum_{\left\{i,j\}\in P(n\right)}A_{i,j}. \end{array}$$

With Equations (41) and (42), the following relationship holds:

(43) $$\begin{array}{c} \sum_{i=1}^{n}\langle R_{i},A\rangle =\frac{2}{(n-3)!!}\sum_{S\in S(n)}\langle S,A\rangle . \end{array}$$

Therefore, the following holds by A∼B and Equation (43):

(44) $$\begin{array}{ccc} \sum_{i=1}^{n}\langle R_{i},A\rangle & = & \frac{2}{(n-3)!!}\sum_{S\in S(n)}\langle S,A\rangle \\ & = & \frac{2}{(n-3)!!}\sum_{S\in S(n)}\langle S,B\rangle \\ & = & \sum_{i=1}^{n}\langle R_{i},B\rangle . \end{array}$$

By Equation (44), we can cancel the second and third terms of (40),

(45) $$\begin{array}{c} \langle S,A^{*}\rangle =\langle S,B\rangle . \end{array}$$

In addition, A∼B holds. Therefore,

(46) $$\begin{array}{c} \forall S\in S\left(n\right),\langle S,A^{*}\rangle =\langle S,B\rangle =\langle S,A\rangle . \end{array}$$

Additionally, the following also holds by A∼B and Equation (44):

(47) $$\begin{array}{ccc} \sum_{j,j\neq i}A_{i,j}^{*} & = & \frac{n-1}{n-2}\langle R_{i},A\rangle +\frac{1}{n-2}\sum_{j,j\neq i}\langle R_{j},A\rangle +\sum_{j,j\neq i}B_{i,j}\\ & & -\frac{n-1}{n-2}\langle R_{i},B\rangle -\frac{1}{n-2}\sum_{j,j\neq i}\langle R_{j},B\rangle \\ & = & \frac{1}{n-2}\left(\sum_{j=1}^{n}\langle R_{j},A\rangle -\sum_{j=1}^{n}\langle R_{j},B\rangle \right)+\langle R_{i},A\rangle \\ & = & \langle R_{i},A\rangle . \end{array}$$

By Equation (47),

(48) $$\begin{array}{c} 1\leq i\leq n,\langle R_{i},A^{*}\rangle =\langle R_{i},A\rangle . \end{array}$$

Therefore, by Equations (46) and (48) and Corollary 1,

(49) $$\begin{array}{c} A=A^{*} \end{array}$$

is valid. That is to say, the following equation holds:

(50) $$\begin{array}{c} \left\{i,j\right\}\in P\left(n\right),A_{i,j}-\frac{1}{n-2}\left(\langle R_{i},A\rangle +\langle R_{j},A\rangle \right)=B_{i,j}-\frac{1}{n-2}\left(\langle R_{i},B\rangle +\langle R_{j},B\rangle \right). \end{array}$$

□

### 3.3. Mean and Covariance

Here, we analyze statistical properties associated with the compatibility matrix and the total compatibility.

We define the mean values of compatibilities and total compatibilities as

$$\begin{array}{ccc} C\in \Omega_{n},\\ \mu_{\mathrm{element}}\left(C\right) & \equiv & \frac{2}{n(n-1)}\sum_{1\leq i<j\leq n}C_{i,j},\\ \mu_{\mathrm{sum}}\left(C\right) & \equiv & \frac{1}{(n-1)!!}\sum_{S\in S(n)}\langle S,C\rangle . \end{array}$$

By Equation (42), $\mu_{\mathrm{sum}}\left(C\right)$ is transformed into

(51) $$\begin{array}{ccc} \mu_{\mathrm{sum}}\left(C\right) & \equiv & \frac{1}{(n-1)!!}\sum_{S\in S(n)}\langle S,C\rangle \\ & = & \frac{1}{n-1}\sum_{1\leq i<j\leq n}C_{i,j}\\ & = & \frac{n}{2}\mu_{\mathrm{element}}\left(C\right) \end{array}$$

where $\mu_{\mathrm{element}}\left(C\right)$ indicates the mean value of the elements of the compatibility matrix *C* and $\mu_{\mathrm{sum}}\left(C\right)$ is the mean of the total compatibility across all possible pairing with respect to the compatibility matrix *C*.

We define the square root of the covariance values for compatibilities and total compatibilities as follows:

(52) $$\begin{array}{ccc} & & \sigma_{\mathrm{element}}\left(A,B\right)\equiv \sqrt{\sum_{1\leq i<j\leq n}\frac{2}{n(n-1)}\left(A_{i,j}-\mu_{\mathrm{element}}\left(A\right)\right)\left(B_{i,j}-\mu_{\mathrm{element}}\left(B\right)\right)},\\ & & \sigma_{\mathrm{sum}}\left(A,B\right)\equiv \sqrt{\frac{1}{(n-1)!!}\sum_{S\in S(n)}\left(\langle S,A\rangle -\mu_{\mathrm{sum}}\left(A\right)\right)\left(\langle S,B\rangle -\mu_{\mathrm{sum}}\left(B\right)\right)}. \end{array}$$

Clearly, $\sigma_{\mathrm{element}}^{2}\left(C,C\right)$ and $\sigma_{\mathrm{sum}}^{2}\left(C,C\right)$ are variance values for compatibilities and total compatibilities when the compatibility matrix is *C*.

Regarding $\sigma_{\mathrm{sum}}^{2}\left(C,C\right)$, the following theorem holds.

**Theorem** **3.** *Let $I_{n}$ be the n×n identity matrix, $J_{n}$ the n×n matrix where all elements are 1, and $C\in \Omega_{n},\hat{C}\equiv C-\mu_{\mathrm{element}}\left(C\right)\left(J_{n}-I_{n}\right)$. Then, the following equation holds:*

(53) $$\begin{array}{c} \sigma_{\mathrm{sum}}^{2}\left(C,C\right)=\frac{n(n-2)}{2(n-3)}\sigma_{\mathrm{element}}^{2}\left(C,C\right)-\frac{1}{\left(n-1)(n-3\right)}\sum_{k=1}^{n}{\langle R_{k},\hat{C}\rangle }^{2}. \end{array}$$

**Proof** **of** **Theorem** **3.** By definition,

$$\begin{array}{ccc} \sigma_{\mathrm{sum}}^{2}\left(C,C\right) & = & \frac{1}{(n-1)!!}\sum_{S\in S(n)}{\left\{\langle S,C\rangle -\mu_{\mathrm{sum}}\left(C\right)\right\}}^{2} \end{array}$$

Using Equation (51),

(54) $$\begin{array}{ccc} \sigma_{\mathrm{sum}}^{2}\left(C,C\right) & = & \frac{1}{(n-1)!!}\sum_{S\in S(n)}{\left\{\langle S,C\rangle -\frac{n}{2}\mu_{\mathrm{element}}\left(C\right)\right\}}^{2} \end{array}$$

Here, the following equation holds:

(55) $$\begin{array}{ccc} \langle S,\hat{C}\rangle & = & \frac{1}{2}{\langle S,\hat{C}\rangle }_{F}\\ & = & \frac{1}{2}{\langle S,C\rangle }_{F}-\frac{1}{2}\mu_{\mathrm{element}}\left(C\right)\langle S,J_{n}-I_{n}\rangle \\ & = & \frac{1}{2}{\langle S,C\rangle }_{F}-\frac{n}{2}\mu_{\mathrm{element}}\left(C\right)\\ & = & \langle S,C\rangle -\frac{n}{2}\mu_{\mathrm{element}}\left(C\right) \end{array}$$

Therefore, by Equations (54) and (55),

(56) $$\begin{array}{ccc} \sigma_{\mathrm{sum}}^{2}\left(C,C\right) & = & \frac{1}{(n-1)!!}\sum_{S\in S(n)}{\left\{\langle S,C\rangle -\frac{n}{2}\mu_{\mathrm{element}}\left(C\right)\right\}}^{2}\\ & = & \frac{1}{(n-1)!!}\sum_{S\in S(n)}{\langle S,\hat{C}\rangle }^{2}\\ & = & \frac{1}{(n-1)!!}\cdot \left(n-3\right)!!\sum_{\left\{i,j\}\in P(n\right)}{\hat{C}}_{i,j}^{2}\\ & & +\frac{1}{(n-1)!!}\cdot \left(n-5\right)!!\sum_{\left\{i,j\}\in P(n\right)}\sum_{\begin{array}{c} \left\{k,l\}\in P(n\right)\\ \{k,l\}\cap \{i,j\}=\emptyset \end{array}}{\hat{C}}_{i,j}{\hat{C}}_{k,l}\\ & = & \frac{1}{n-1}\sum_{\left\{i,j\}\in P(n\right)}{\hat{C}}_{i,j}^{2}+\frac{1}{\left(n-1)(n-3\right)}\sum_{\left\{i,j\}\in P(n\right)}\sum_{\begin{array}{c} \left\{k,l\}\in P(n\right)\\ \{k,l\}\cap \{i,j\}=\emptyset \end{array}}{\hat{C}}_{i,j}{\hat{C}}_{k,l}\\ & = & \frac{1}{n-1}\sum_{\left\{i,j\}\in P(n\right)}{\hat{C}}_{i,j}^{2}+\frac{1}{\left(n-1)(n-3\right)}\sum_{\left\{i,j\}\in P(n\right)}{\hat{C}}_{i,j}\sum_{\begin{array}{c} \left\{k,l\}\in P(n\right)\\ \{k,l\}\cap \{i,j\}=\emptyset \end{array}}{\hat{C}}_{k,l}. \end{array}$$

Here, we focus on $\sum_{\begin{array}{c} \left\{k,l\}\in P(n\right)\\ \{k,l\}\cap \{i,j\}=\emptyset \end{array}}{\hat{C}}_{k,l}$. This term is transformed as follows:

(57) $$\begin{array}{ccc} \sum_{\begin{array}{c} \left\{k,l\}\in P(n\right)\\ \{k,l\}\cap \{i,j\}=\emptyset \end{array}}{\hat{C}}_{k,l} & = & {\hat{C}}_{i,j}+\sum_{\left\{k,l\}\in P(n\right)}{\hat{C}}_{k,l}-\sum_{k,k\neq i}{\hat{C}}_{i,k}-\sum_{k,k\neq j}{\hat{C}}_{j,k}\\ & = & {\hat{C}}_{i,j}-\langle R_{i},\hat{C}\rangle -\langle R_{j},\hat{C}\rangle +\sum_{\left\{k,l\}\in P(n\right)}{\hat{C}}_{k,l}\\ & = & {\hat{C}}_{i,j}-\langle R_{i},\hat{C}\rangle -\langle R_{j},\hat{C}\rangle +\sum_{\left\{k,l\}\in P(n\right)}\left(C_{k,l}-\mu_{\mathrm{element}}\left(C\right)\right)\\ & = & {\hat{C}}_{i,j}-\langle R_{i},\hat{C}\rangle -\langle R_{j},\hat{C}\rangle +\left(\sum_{\left\{k,l\}\in P(n\right)}C_{k,l}\right)-\frac{n(n-1)}{2}\mu_{\mathrm{element}}\left(C\right)\\ & = & {\hat{C}}_{i,j}-\langle R_{i},\hat{C}\rangle -\langle R_{j},\hat{C}\rangle . \end{array}$$

Then, using this formula,

(58) $$\begin{array}{ccc} & & \sum_{\left\{i,j\}\in P(n\right)}{\hat{C}}_{i,j}\sum_{\begin{array}{c} \left\{k,l\}\in P(n\right)\\ \left\{k,l\}\neq \{i,j\right\} \end{array}}{\hat{C}}_{k,l}\\ & & =\sum_{\left\{i,j\}\in P(n\right)}{\hat{C}}_{i,j}\left({\hat{C}}_{i,j}-\langle R_{i},\hat{C}\rangle -\langle R_{j},\hat{C}\rangle \right)\\ & & =\sum_{\left\{i,j\}\in P(n\right)}{\hat{C}}_{i,j}^{2}-\sum_{\left\{i,j\}\in P(n\right)}{\hat{C}}_{i,j}\left(\langle R_{i},\hat{C}\rangle +\langle R_{j},\hat{C}\rangle \right)\\ & & =\sum_{\left\{i,j\}\in P(n\right)}{\hat{C}}_{i,j}^{2}-\sum_{i=1}^{n}\sum_{j\neq i}{\hat{C}}_{i,j}\langle R_{i},\hat{C}\rangle \\ & & =\sum_{\left\{i,j\}\in P(n\right)}{\hat{C}}_{i,j}^{2}-\sum_{i=1}^{n}{\langle R_{i},\hat{C}\rangle }^{2}. \end{array}$$

By Equations (56) and (58), the following equation holds:

(59) $$\begin{array}{ccc} \sigma_{\mathrm{sum}}^{2}\left(C,C\right) & = & \frac{n-2}{\left(n-1)(n-3\right)}\sum_{\left\{i,j\}\in P(n\right)}{\hat{C}}_{i,j}^{2}-\frac{1}{\left(n-1)(n-3\right)}\sum_{k=1}^{n}{\langle R_{k},\hat{C}\rangle }^{2}\\ & = & \frac{n(n-2)}{2(n-3)}\sigma_{\mathrm{element}}^{2}\left(C,C\right)-\frac{1}{\left(n-1)(n-3\right)}\sum_{k=1}^{n}{\langle R_{k},\hat{C}\rangle }^{2}. \end{array}$$

Therefore, the theorem holds. □

## 4. Variance Optimization

This section examines the performance enhancement from deriving a pairing that yields higher total compatibility by exploiting the algebraic structures identified in the previous section. We first show that the variance of the elements in a compatibility matrix affects the performance of the heuristic algorithm proposed in our previous study. Then we propose the transformation of a compatibility matrix to another one that minimizes the variance while ensuring that the total compatibility is maintained.

### 4.1. Performance Degradation through the Observation Phase

In our previous study [17], we proposed an algorithm for recognizing the compatibilities among elements through multiple measurements of total compatibility. To summarize, we estimate the compatibility matrix denoted by $\tilde{C}\in \Omega_{n}$, which is given by

(60) $$\begin{array}{ccc} & & C\in \Omega_{n},\\ & & {\tilde{C}}_{i,j}=\left\{\begin{array}{c} 0\;\mathrm{if}\;1\in \{i,j\}\\ C_{i,j}-C_{1,i}-C_{1,j}+\frac{2}{n-2}\sum_{k=2}^{n}C_{1,k}\;\mathrm{otherwise}. \end{array}\right. \end{array}$$

This $\tilde{C}\in \Omega_{n}$ is one of the elements in the equivalence class. That is, $C∼\tilde{C}$ holds. By this property and Equation (60), the dimension of ${\left\{S\right\}}_{S\in S(n)}$ is given by (n−1)(n−2)/2, which we refer to as $L_{\min}\left(n\right)$. This means that the number of observations required to grasp the compatibilities through an observation phase is $L_{\min}\left(n\right)$.

Indeed, our previous study proposed an observation algorithm which needs $O(n^{2})$ measurements. We have also confirmed numerically that the observation strategy provides a compatibility matrix, which is in the equivalence class of the ground-truth compatibility matrix $C^{g}$. In the numerical studies, the elements of the ground-truth compatibility matrix, $C_{i,j}^{g}$, were specified by uniformly distributed random numbers in the range of [0,1].

However, finding a pairing yielding a greater total compatibility becomes difficult based on $C^{e}$, including the above-mentioned $\tilde{C}$, even though $C^{e}$ is in the equivalence class where the ground-truth compatibility $C^{g}$ is included. In searching for a better pairing, we use a heuristic algorithm, which is named Pairing-2-opt [17]. We consider the difficulty comes from the fact that the variance of the elements of the compatibility matrix $\sigma_{\mathrm{element}}^{2}\left(C^{e},C^{e}\right)$ would be larger than those of $\sigma_{\mathrm{element}}^{2}\left(C^{g},C^{g}\right)$, which is highly likely to cause the combining algorithm to become stuck in a local minimum.

Hence, our idea is to find a compatibility matrix *X* which is in the same equivalence class of matrix *C*

(61) $$\begin{array}{c} \forall S\in S(n),\langle S,X\rangle =\langle S,C\rangle \end{array}$$

while simultaneously minimizing the variance of the elements of $\sigma_{\mathrm{element}}^{2}\left(X,X\right)$.

### 4.2. Transforming the Compatibility Matrix with Minimized Variance

We solve the following optimization problem:

(62) $$\begin{array}{ccc} \min: & & \sigma_{\mathrm{element}}^{2}\left(X,X\right),\\ \mathrm{subject}\;\mathrm{to}: & & X,C\in \Omega_{n},C\;\mathrm{is}\;\mathrm{fixed},\\ & & X∼C. \end{array}$$

By Theorem 3 and $\sigma_{\mathrm{sum}}^{2}\left(X,X\right)=\sigma_{\mathrm{sum}}^{2}\left(C,C\right)$, we transform this problem into the following form:

(63) $$\begin{array}{ccc} \min: & & \sum_{k=1}^{n}{\langle R_{k},\hat{X}\rangle }^{2},\\ \mathrm{subject}\;\mathrm{to}: & & X,C\in \Omega_{n},C\;\mathrm{is}\;\mathrm{fixed},\\ & & X∼C,\\ & & \hat{X}\equiv X-\mu_{\mathrm{element}}\left(X\right)\left(J_{n}-I_{n}\right). \end{array}$$

The optimal solution for this problem holds because the sum of squares is minimized when all values are 0:

(64) $$\begin{array}{ccc} & & 1\leq k\leq n,\langle R_{k},\hat{X}\rangle =0. \end{array}$$

Hence, the following equation is derived:

(65) $$\begin{array}{c} 1\leq k\leq n,\langle R_{k},X\rangle =\left(n-1\right)\mu_{\mathrm{element}}\left(C\right). \end{array}$$

By Equation (65) and Theorem 2, the optimal solution is represented as follows:

(66) $$\begin{array}{ccc} & & X_{i,j}=\frac{2(n-1)}{n-2}\mu_{\mathrm{element}}\left(C\right)+C_{i,j}-\frac{1}{n-2}\left(\langle R_{i},C\rangle +\langle R_{j},C\rangle \right). \end{array}$$

Thus, the compatibility matrix with minimal variance is derived. In addition, this discussion and solution mean that the optimal-variance solution is unique with respect to the equivalence class.

## 5. Simulation

In this section, we evaluate the performance of the proposed method on the pairing optimization problem. There are two important points that should be clarified through the simulations. One is to quantitatively evaluate the performance reduction of the combining algorithm proposed in the previous study, based on the observation phase. The other is to demonstrate the performance enhancement due to the variance optimization discussed in Section 4.

### 5.1. Setting

We configure the ground-truth compatibility matrix $C^{g}\in \Omega_{n}$ with two different distributions. The first is the uniform distribution:

(67) $$\begin{array}{c} \forall \left\{i,j\right\}\in P,C_{i,j}^{g}∼U\left(0,1\right). \end{array}$$

Here, we denote the uniform distribution between 0 and 1 as U(0,1). The second distribution is the Poisson distribution:

(68) $$\begin{array}{c} \forall \left\{i,j\right\}\in P,C_{i,j}^{g}∼Poisson\left(1\right). \end{array}$$

Here, we denote the Poisson distribution whose mean is λ as Poisson(λ). In the numerical simulation, the number of elements in the system *n* varied from 100 to 1000 in intervals of 100. For each *n*, we conducted 100 trials with different randomly generated ground-truth compatibility matrices $C^{g}$ based on Equation (67) or Equation (68). We quantified the performance for each derived pairing S∈S(n) by $2\langle S,C^{g}\rangle /n$ and evaluated its average over 100 trials for each value of *n*.

### 5.2. Simulation Flow

The ground-truth compatibility matrix $C^{g}$ is transformed into $C^{e_{1}}$ by the observation algorithm based on Equation (60). The variance optimization transforms $C^{e_{1}}$ into $C^{e_{2}}$. The combining algorithm, which is called PNN+p2-opt [17], yields a pairing with the intention of achieving higher total compatibility. The exchange limit *l* is an internal parameter in PNN+p2-opt. This determines the number of maximum trials, and is set to 600 in the present study.

**Figure 2:**
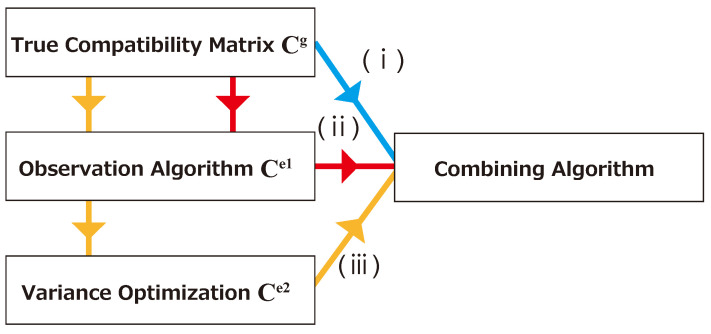
Schematic illustration of the three heuristic pairing optimization algorithms tested in the simulation. Case (i) (blue) applies the combining algorithm directly to the ground-truth compatibility matrix $C^{g}$. Case (ii) (red) first applies the observation algorithm to obtain an estimated compatibility matrix $C^{e_{1}}$, followed by the combining algorithm. Case (iii) (yellow) first estimates the compatibility from observation ($C^{e_{1}}$), followed by the variance optimization ($C^{e_{2}}$), and then executes the combining algorithm.

We evaluated the performance on the basis of $C^{g}$, $C^{e_{1}}$, and $C^{e_{2}}$, as shown in flows (i), (ii), and (iii), respectively, in Figure 2.

### 5.3. Performance

**Figure 3:**
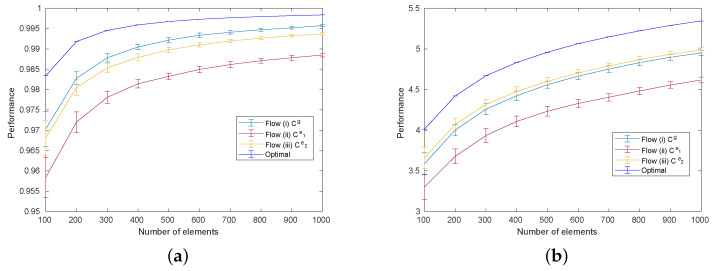
Comparison of the achieved total compatibility for Flows (i), (ii), and (iii), as described in the caption for Figure 2. Each graph shows the mean and standard deviation of the performance of 100 different compatibility matrices with each given number of elements, simulated under (**a**) uniform distributions and (**b**) Poisson distributions.

The blue, red, and yellow curves in Figure 3 demonstrate the performance of cases (i), (ii), and (iii), respectively, as a function of the number of elements for the uniform distribution (Figure 3a) and the Poisson distribution (Figure 3b). For the uniform distributed ground-truth we observe that the performance of case (ii) is inferior to that of case (i), demonstrating the performance degradation by the transformation from $C^{g}$ to $C^{e_{1}}$ through observation. Furthermore, the performance of case (iii) is enhanced compared with that of case (ii), which confirms the performance gain from variance optimization. The results differ for the Poisson distribution. Here, the performance of case (iii) is higher than case (i). That is, for the Poisson case the variance optimization (Flow (iii)) not only counteracted the performance loss of the observation algorithm (Flow (ii)), but actually enhanced the performance compared to the ground truth matrix $C^{g}$ (Flow (i)). Further numerical tests revealed that the relationship of performances for a Gaussian distribution are similar to those for the uniform distribution. Conversely, the performance for a binary distribution hardly differed between any of the algorithms.

**Figure 4:**
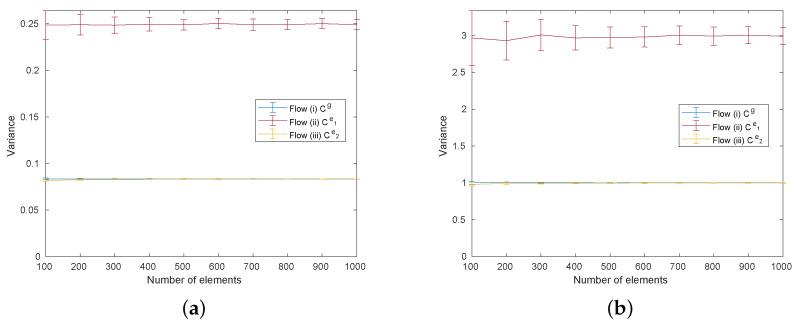
Comparison of the variance of the compatibility matrices of $C^{g}$, $C^{e_{1}}$, $C^{e_{2}}$ as a function of the number of elements in the system under (**a**) uniform distributions and (**b**) Poisson distributions.

The variance of $C^{g}$, $C^{e_{1}}$, and $C^{e_{2}}$ are evaluated as shown in Figure 4 as a function of the number of elements. We clearly observe that the variance of $C^{e_{1}}$ is higher than $C^{g}$ while the variance of $C^{e_{2}}$ becomes comparable to the ground-truth case $C^{g}$ for both the uniform and Poisson distributions.

From these numerical results, we can conclude that the variance optimization minimizes the variance and enhances the performance of the achieved total compatibility. It is worth noting that the performance with the uniform distribution after variance optimization is still lower than the case based on the ground-truth matrix $C^{g}$, as observed in Figure 3a. This occurs because the variance optimization algorithm does not transform $C^{e_{1}}$ to the original compatibility matrix $C^{g}$. In other words, there exist additional factors that influence the performance of the combining algorithm that are related to the compatibility distribution. The distribution of the original compatibility $C^{g}$ (uniform distribution) is seemingly beneficial for the performance of the heuristic combining algorithm, even when compared to the compatibility matrix with minimum variance $C^{e_{2}}$.

## 6. Conclusions

One of the most challenging issues in the pairing problem is how to understand the underlying compatibilities among the elements under study. An accurate and efficient approach is essential for practical applications such as wireless communications and online social networks. This study reveals several algebraic structures in the pairing optimization problem.

We introduce an equivalence class in the compatibility matrices, containing matrices that yield the same total compatibility although the matrices themselves differ. This can also be expressed through a conserved value or invariance in the equivalence class. Based on such insights, we propose a transformation of the initially estimated compatibility matrix to another form that minimizes the variance of the elements. We demonstrate that the highest total compatibility found heuristically is improved significantly with the proposed transformation relative to the direct approach.

In the future, the proposed algorithm may be applied to bipartite matching and assignment problems, for example. Therefore, if the compatibility between elements that should not be paired is set to a negative value with a relatively large absolute value, we may solve the problem heuristically. Hence, the variance optimization proposed in this study may aid in performance enhancement.

## Figures and Tables

**Figure A1:**
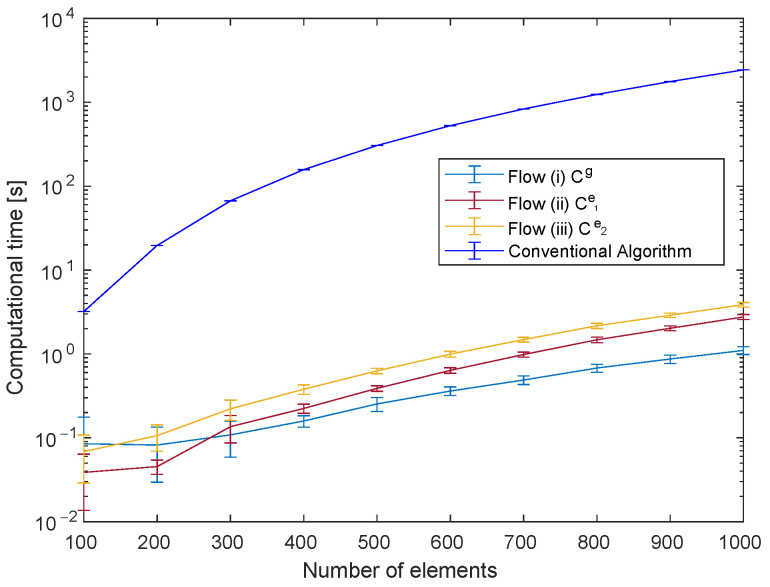
Comparison of computational time between different four algorithms, which are case (i), (ii), (iii) and the conventional MWM algorithm.

## Data Availability

The data that support the findings of this study are available from the corresponding author upon reasonable request.

## Appendix A. Matrix Form of Conserved Quantities

In Theorem 2, the following values are conserved in the same equivalence class.

(A1) $$\begin{array}{c} \forall \left\{i,j\right\}\in P\left(n\right),C_{i,j}-\frac{1}{n-2}\left(\langle R_{i},C\rangle +\langle R_{j},C\rangle \right). \end{array}$$

We can transform Equation (A1) into the following form using the Hadamard product ∘.

(A2) $$\begin{array}{c} C-\frac{1}{n-2}\left(J_{n}-I_{n}\right)\circ \left(J_{n}C+CJ_{n}\right). \end{array}$$

Therefore, the following equation holds:

(A3) $$\begin{array}{ccc} & & A∼B\;\mathrm{if}\mathrm{and}\mathrm{only}\mathrm{if}\;\\ & & A-\frac{1}{n-2}\left(J_{n}-I_{n}\right)\circ \left(J_{n}A+AJ_{n}\right)=B-\frac{1}{n-2}\left(J_{n}-I_{n}\right)\circ \left(J_{n}B+BJ_{n}\right). \end{array}$$

## Appendix B. Computational Time

We compared the computational time of four different algorithms in the new Figure A1 in the new version of the manuscript. Three of them are for the cases from Figure 2. For cases (ii) and (iii), the computational time also includes the time needed for variance optimization. We compared them to a conventional MWM algorithm whose code (https://jp.mathworks.com/matlabcentral/fileexchange/42827-weighted-maximum-matching-in-general-graphs (accessed on 10 January 2023)) was developed and distributed by Daniel R. Saunders (http://danielrsaunders.com (accessed on 10 January 2023)). This conventional algorithm is based on “Efficient algorithms for finding maximum matching in graphs” by Zvi Galil [32]. The number of elements changes from 100 to 1000. One hundred different compatibility matrices were simulated and averaged to obtain the computational time.

Figure A1 shows that, as expected, the PNN+p2-opt algorithm is significantly faster than the conventional algorithm, and Flow (i), Flow (ii), Flow (iii) work faster in this order. These computational times can be explained as follows: First, PNN+p2-opt is heuristic and a $O(n^{2})$ algorithm. Therefore, PNN+p2-opt is significantly faster than the conventional MWM algorithm that aims to find the absolute best solution. Second, we count the computational time, including the variance optimization procedure. The variance optimization takes some time, so the computational time of flow (ii) and flow (iii) is longer than flow (i). Third, flow (ii) has a tendency to become stuck in local minima, resulting in less computational time than flow (iii), due to the faster termination of the p2-opt algorithm.

In the future, the comparison to machine-learning-based methods such as the one proposed in Ref. [16] is of great interest. However, at this point, it is unclear how to conduct a fair comparison, as the ML-based algorithm requires extensive training on multiple examples before it is able to solve the problem. Nevertheless, as machine learning is a rapidly evolving field, it is possible that ML-based algorithms specialized for the pairing problem could be developed in the near future.
